# Gene function classification using Bayesian models with hierarchy-based priors

**DOI:** 10.1186/1471-2105-7-448

**Published:** 2006-10-12

**Authors:** Babak Shahbaba, Radford M Neal

**Affiliations:** 1 Dept. of Public Health Sciences, Biostatistics, University of Toronto, Toronto, Ontario, Canada; 2 Dept. of Statistics and Dept. of Computer Science, University of Toronto, Toronto, Ontario, Canada

## Abstract

**Background:**

We investigate whether annotation of gene function can be improved using a classification scheme that is aware that functional classes are organized in a hierarchy. The classifiers look at phylogenic descriptors, sequence based attributes, and predicted secondary structure. We discuss three Bayesian models and compare their performance in terms of predictive accuracy. These models are the ordinary multinomial logit (MNL) model, a hierarchical model based on a set of nested MNL models, and an MNL model with a prior that introduces correlations between the parameters for classes that are nearby in the hierarchy. We also provide a new scheme for combining different sources of information. We use these models to predict the functional class of Open Reading Frames (ORFs) from the *E. coli *genome.

**Results:**

The results from all three models show substantial improvement over previous methods, which were based on the C5 decision tree algorithm. The MNL model using a prior based on the hierarchy outperforms both the non-hierarchical MNL model and the nested MNL model. In contrast to previous attempts at combining the three sources of information in this dataset, our new approach to combining data sources produces a higher accuracy rate than applying our models to each data source alone.

**Conclusion:**

Together, these results show that gene function can be predicted with higher accuracy than previously achieved, using Bayesian models that incorporate suitable prior information.

## Background

Annotating genes with respect to the function of their proteins is essential for understanding the wealth of genomic information now available. A direct approach to identifying gene function is to eliminate or inhibit expression of a gene and observe any alteration in the phenotype. However, analysis of all genes for all possible functions is not feasible at present. Statistical methods have therefore been employed for this purpose. One statistical approach attempts to predict the functional class of a gene based on similar sequences for which the function is known. The similarity measures used for this task are produced by computer algorithms that compare the sequence of interest against all other sequences with known function. Two commonly used algorithms are BLAST [1] and FASTA [2].

A problem with using such similarity measures is that a gene's function cannot be predicted when no homologous gene of known function exists. To improve the quality and coverage of prediction, other sources of information can be used. For example, King *et al*. [3] used a variety of protein sequence descriptors, such as residue frequency and the predicted secondary structure (the structure of hydrogen bonding between different residues within a single polypeptide chain). DeRisi *et al*. [4], Eisen *et al*., [5] and Brown *et al*. [6] used gene expression data, on the assumption that similarly expressed genes are likely to have similar function. Marcotte *et al*. [7] recommended an alternative sequence-based approach that regards two genes as similar if they are together in another genome. Deng *et al*. [8] predict the function of genes from their network of physical interactions. To address some of the problems associated with similarity-based methods, such as their non-robustness to variable mutation rates [9,10], annotation of protein sequences using phylogenetic information has been suggested by some authors (e.g., [5,11,12]). In this approach, the evolutionary history of a specific protein, captured by a phylogenetic tree, is used for annotating that protein [5].

The above-mentioned sources of data can be used separately, or as proposed by several authors (e.g., [3,13,14]), they can be combined within a predictive model. A variety of statistical and machine learning techniques for making such predictions have been used in functional genomics. These include neighbourhood-count methods [15], support vector machines [6], and Markov random fields [8]. A common feature of these models is that they treat classes as unrelated entities without any specific structure.

The assumption of unrelated classes is not always realistic. As argued by Rison *et al*. [16], in order to understand the overall mechanism of the whole genome, the functional classes of genes need to be organized according to the biological processes they perform. For this purpose, many functional classification schemes have been proposed for gene products. The first such scheme was recommended by Riley [17] to catalogue the proteins of *Escherichia coli*. Since then, there have been many attempts to provide a standardized functional annotation scheme with terms that are not limited to certain types of proteins or to specific species. These schemes usually have a hierarchical structure, which starts with very general classes and becomes more specific in lower levels of the hierarchy. In some classification hierarchies, such as the Enzyme Commission (EC) scheme [18], levels have semantic values [16]. For example, the first level of the EC scheme represents the major activities of enzyme like "transferaces" or "hydrolases". In some other schemes, like the ones considered here, the levels do not have any uniform meaning. Instead, each division is specific to the parent nodes. For instance, if the parent includes "metabolism" functions, the child nodes could be the metabolism of "large" or "small" molecules. Rison *et al*. [16] surveyed a number of these structures and compared them with respect to their resolution (total number of function nodes), depth (potential of the scheme for division into subsets) and breadth (number of nodes at the top level).

All these hierarchies provide additional information that can be incorporated into the classification model. The importance of using the hierarchy in classification models has been emphasized by many authors (e.g., [19-21]). One approach for modelling hierarchical classes is to decompose the classification model into nested models, one for each node of the hierarchy. Goodman [22] showed that using nested models can significantly reduce the training time of maximum entropy-based language models and results in slightly lower perplexities. He illustrated his approach using a word labelling problem, and recommended that instead of predicting words directly, we first predict the class to which the word belongs, and then predict the word itself. Weigend *et al*. [23] also used a two-level hierarchical model for document classification. They evaluated their model on the Reuters-22173 corpus and showed significant improvement, especially for rare classes. For text classification, McCallum *et al*. [24] proposed a hierarchical naive Bayes model that smoothes parameter estimates of a child node by shrinking toward its parents in order to obtain more robust parameter estimates. More recently, new hierarchical classification models based on large margin principles, specifically support vector machines (SVM), have been proposed [25-29]. Dekel *et al*. [26] introduced a large margin hierarchical classification model that uses the sum of parameters along the tree for classifying cases to the end nodes. These parameters are estimated based on a set of classifiers that assign cases to the intermediate nodes. Cai and Hoffmann [27] suggested a similar approach based on the generalization of multiclass SVM.

Many approaches to using the hierarchy of gene functions have been proposed. Eisner *et al*. [30] build multiple binary classifiers with training sets modified according to Gene Ontology (GO). For each classifier associated with a node, they regard a gene as a positive example if it belongs to that node, and as a negative example if it does not belong to the node, or to the node's ancestors and descendants.

Barutcuoglu *et al*. [31] also use a set of independent classifiers, whose predictions are combined using a Bayes network defined based on the GO hierarchy. In the methods recommended by both [30] and [31], the individual classifiers are built independently. Although the classifiers are modified to become consistent, it is more natural to model classes simultaneously. Many authors have shown that learning a set of related tasks at the same time will improve the performance of models (e.g., [32,33]). King *et al*. [3] attempted to use the additional information from the hierarchical structure of gene functional classes by simply using different decision tree models for each level of the hierarchy. Clare and King [34] investigated a modified decision tree model, in which assignment of a functional class to a node in the decision tree implies membership of all its parent classes. They evaluated this method based on *Saccharomyces cerevisiae *data and found that the modified version is sometimes better than the non-hierarchical model and sometimes worse. Blockeel *et al*. [35] suggested an alternative modification of decision trees for hierarchical classification models. Their model uses a distance-based measure, where distances are derived from the hierarchy. Struyf *et al*. [36] followed the same idea but advocated a different distance measure, which is easier to interpret and is guaranteed to be positive. They evaluated their approach based on different datasets available for *Saccharomyces cerevisiae*, and showed that their model has better precision than the hierarchical C4.5 model proposed by [34].

In a previous paper [37], we introduced an alternative Bayesian framework for modelling hierarchical classes. This method, henceforth called corMNL, uses a Bayesian form of the multinomial logit model (MNL), with a prior that introduces correlations between the parameters for classes that are nearby in the tree. We also discussed an alternative hierarchical model that uses the hierarchy to define a set of nested multinomial logit models, which we refer to as treeMNL. In this paper, we apply these methods (described further below in the methods section) to the gene function classification problem.

## Results and discussion

We used our Bayesian MNL, treeMNL and corMNL models to predict the functional class of Open Reading Frames (ORFs) from the *E. coli *genome. *E. coli *is a good organism for testing our method since many of its gene functions have been identified through direct experiments. We used the pre-processed data provided by [3]. This dataset contains 4289 ORFs identified by [38]. Only 2122 of these ORFs, for which the function was known in 2001, are used in our analysis. The functional hierarchy for these proteins is provided by [39]. This hierarchy has three levels, with the most general classes at level 1 and the most specific classes at level 3. For example, lipoate-protein ligase A (lplA) belongs to class 'Macromolecule metabolism' at level 1, to class 'Macromolecule synthesis, modification' at level 2, and to class 'Lipoprotein' at level 3. After excluding categories 0 and 7 at level 1, the data we used had 6 level 1 categories, 20 level 2 categories, and 146 level 3 categories.

Since 2001 many additional gene functions have been determined by direct experiment (see [40]). However, we use the same dataset as [3], with the same split of data into the training set (1410 ORFs) and test set (712 ORFs), in order to produce comparable results. King *et al*. [3] further divided the training set into two subsets and used one subset as validation data to select a subset of rules from those produced by the C5 algorithm based on the other part of the training set. Our Bayesian methods do not require a validation set, so we did not subdivided the training set.

The covariates are based on three different sources of information: phylogenic descriptors, sequence based attributes, and predicted secondary structure. Following [3], we refer to these three sources of data as SIM, SEQ and STR respectively. Attributes in SEQ are largely based on composition of residues (i.e., the number of residues of type *R*) and of pairs of residues (i.e., the number of residue pairs of types *R *and *S*) in a sequence. There are 933 such attributes (see Table 1 in [3]). Information in SIM (see Table 2 in [3]) and STR (see Table 3 in [3]) is derived based on a PSI-BLAST (position-specific iterative BLAST) search with parameters *e *= 10, *h *= 0.0005, *j *= 20 from NRProt 05/10/99 database. King *et al*. [3] used the Inductive Logic Programming (ILP) algorithm known as Warmr [41] to produce binary attributes based on the identified frequent patterns (1 if the pattern is present and 0 otherwise) in SIM and STR data. The rules created by Warmr and their corresponding attributes can be found at [42]. There are 13799 such attributes generated for SIM and 18342 attributes for STR. As described below in the methods section, we reduced the dimensionality for each dataset using Principal Component Analysis (PCA). We used 100 components for SEQ, 100 components for STR, and 150 components for SIM.

**Table 1:** Comparison of models based on their predictive accuracy (%) using each data source separately.

Accuracy (%)	SEQ			STR			SIM		
	Level 1	Level 2	Level 3	Level 1	Level 2	Level 3	Level 1	Level 2	Level 3
Baseline	42.56	21.21	8.15	42.56	21.21	8.15	42.56	21.21	8.15
MNL	60.25	33.99	20.93	50.98	25.14	15.87	69.10	45.79	30.76
treeMNL	59.27	34.13	18.26	52.67	27.39	16.29	67.70	45.93	30.34
corMNL	**61.10**	**35.96**	**21.21**	**52.81**	**27.95**	**16.71**	**70.51**	**47.19**	**30.90**

**Table 2:** Comparison of models based on their predictive accuracy (%) for specific coverage (%) provided in parenthesis. The C5 results and the coverage values are from [3].

Accuracy (%)	SEQ			STR			SIM		
	Level 1	Level 2	Level 3	Level 1	Level 2	Level 3	Level 1	Level 2	Level 3
	(20)	(18)	(4)	(10)	(1)	(5)	(29)	(26)	(16)
C5	64	63	41	59	44	17	75	74	69
MNL	81	79	88	**83**	**100**	67	96	**90**	**84**
treeMNL	81	76	70	70	86	69	95	87	84
corMNL	**84**	**82**	**89**	**83**	**100**	**73**	**97**	**90**	82

**Table 3:** Accuracy (%) of models on the combined dataset with and without separate scale parameters. Results using SIM alone are provided for comparison.

Accuracy (%)	SIM only			Combined dataset single scale parameter			Combined dataset separate scale parameters		
	Level 1	Level 2	Level 3	Level 1	Level 2	Level 3	Level 1	Level 2	Level 3
MNL	69.10	45.79	30.76	69.66	48.88	32.02	70.65	**49.16**	33.71
treeMNL	67.70	45.93	30.34	68.26	46.63	30.34	68.82	46.63	31.74
corMNL	**70.51**	**47.19**	**30.90**	**71.49**	**49.30**	**32.87**	**72.75**	**49.16**	**34.41**

Table 1 compares the three models with respect to their accuracy of prediction at each level of the hierarchy. In this table, level 1 corresponds to the top level of the hierarchy, while level 3 refers to the most detailed classes (i.e., the end nodes). For level 3, we use a simple 0/1 loss function and minimize the expected loss by assigning each test case to the end node with the highest posterior predictive probability. We could use the same predictions for measuring the accuracy at levels 1 and 2, but to improve accuracy, we instead make predictions based on the total posterior predictive probability of nodes at level 1 and level 2.

To provide a baseline for interpreting the results, for each task we present the performance of a model that ignores the covariates and simply assigns genes to the most common category at the given level in the training set.

As we can see in Table 1, corMNL outperforms all other models. For the SEQ dataset, MNL performs better than treeMNL. Compared to MNL, the corMNL model achieves a slightly better accuracy at level 3 and more marked improvements at level 1 and level 2. For the STR dataset, both hierarchical models (i.e., treeMNL and corMNL) outperform the non-hierarchical MNL. For this dataset, corMNL has a slightly better performance than treeMNL. For the SIM dataset, the advantage of using the corMNL model is more apparent in the first and second levels.

For analysing these datasets, King *et al*. [3] used a decision tree model based on the C5 algorithm. They selected sets of rules that had an accuracy of at least 50% with the coverage of at least two correct examples in the validation set. In Table 2, we compare the accuracy of our models to those of [3]. In order to make the results comparable, we used the same coverage values as they used. Coverage is defined as the percentage of test cases for which we make a confident prediction. In a decision tree model, these test cases can be chosen by selecting rules that lead to a specific class with high confidence. For our models, we base confidence on posterior predictive probability, which is defined as the expected probability of each class with regard to the posterior distribution of model parameters. We assign each test case to a class with the maximum posterior predictive probability. The higher this probability, the more confident we are in classifying the case. We rank the test cases based on how high the highest probability is, and for a coverage of *g*, we classify only the top *g *percent of genes. In Table 2, the coverage values are given in parenthesis. All three of our models discussed here substantially outperform the decision tree model. Overall, corMNL has better performance than MNL and treeMNL.

In an attempt to improve predictive accuracy, King *et al*. [3] combined the three datasets (SEQ, STR and SIM). Although one would expect to obtain better predictions by combining several sources of information, their results showed no additional benefit compared to using the SIM dataset alone. We also tried combining datasets in order to obtain better results. Initially, we used the principal components which we found individually for each dataset, and kept the number of covariates contributed from each data source the same as before. Principal components from each dataset were scaled so that the standard deviation of the first principal component was 1. We did this to make the scale of variables from different data sources comparable while preserving the relative importance of principal components within a dataset.

Using the combined dataset, all our models provided better predictions, although the improvement was only marginal for some predictions. We speculated that some of the covariates may become redundant after combining the data (i.e., are providing the same information). One may often obtain better results by removing redundancy and reducing the number of covariates. To examine this idea, we kept the number of principal components from SIM as before (i.e., 150) but only used the first 25 principal components from SEQ and STR. The total number of covariates was therefore 200. Reducing the number of covariates from SEQ and STR may also prevent them from overwhelming the covariates from SIM, which is the most useful single source. This strategy led to even higher accuracy rates compared to when we used the SIM dataset alone. The results are shown in Table 3 (middle section). It is worth noting that when SEQ and STR are used alone, using 25 principal components (rather than 100 before) results in lower accuracy (results not shown).

To improve the models even further, we tried an alternative strategy in which different sources of data are combined such that their relative weights are automatically adjusted. As we can see in Table 3 (right section), this strategy, which is described in more detail in the methodology section, resulted in further improvements in the performance of the models. We also examined this approach with larger numbers of covariates. We found that when we increased the number of principal components for SEQ and STR back to the original 100, the accuracy of predictions mostly remained the same, though a few dropped slightly.

In practice, we might be most interested in genes whose function can be predicted with high confidence. There is a trade-off between predictive accuracy and the percentage of the genes we select for prediction (i.e., coverage). Table 4 shows this trade-off for results on the test set from the corMNL model applied to the combined dataset. In this table, the accuracy rates for different coverage values are provided. As we can see, our model can almost perfectly classify 10% of the genes in the test set.

**Table 4:** Predictive accuracy (%) for different coverage values (%) of the corMNL model using all three sources with separate scale parameters.

Accuracy (%)	Coverage (%)					
	*5*	*10*	*20*	*50*	*90*	*100*
Level 1	100	98	96	92	76	73
Level 2	100	98	96	71	53	49
Level 3	100	97	80	52	36	34

Finally, we trained the corMNL model on all ORFs with known function to annotate the function of unknown ORFs. Many of these ORFs, whose function was previously unknown, have been recently annotated using direct biological experiments. However, since the functional ontology of *E. coli *genome (provided by the Riley group) has changed over time, it is not possible to compare our results directly. King *et al*. [40] also faced this problem. They evaluated their predictions manually for a subset of ORFs. We present our predictions for the same set of ORFs [see Additional file 1 ]. We use the MultiFun Classification System [43] to obtain the function(s) associated with each ORF through direct experiments.

For many of these ORFs, our prediction is closely related to the confirmed function. For example, we classified *yojH *(b2210) hierarchically as "Metabolism of small molecules" at the first level, "Degradation of small molecules" at the second level, and "Carbon compounds" at the third level. Through a direct experiment, the function of this gene was classified as "Metabolism", "Energy metabolism (carbon)", and "Tricarboxylic acid cycle", at levels 1, 2, and 3 respectively. In some cases, such as *ybhO *(b0789), there is an exact match between our prediction (Macromolecule metabolism : Macromolecule synthesis : Phospholipids) and the function provided by MultiFun (Metabolism : Macromolecule (cellular constituent) biosynthesis : Phospholipid). For some other cases, although our prediction does not exactly match the functions provided by MultiFun Classification System, the results are comparable up to the first or second level of the hierarchy. For example, we predicted that *ydeD *(b1533) is "Transport/binding proteins" and belongs to "ABC superfamily". Direct experiment also show that in fact this gene does belongs to the "Transport" group, however, it is more specifically in the "Major Facilitator Superfamily" (MFS) class instead of ABC. The comparison of our predictions with MultiFun Classification System is not always as straightforward as the examples provided above. For instance, we predicted *bfd *(b3337) to be in the "Metabolism of small molecules : Energy metabolism, carbon : Anaerobic respiration" categories. The hierarchical function provided by MultiFun is "Cell processes : Adaptation to stress : Fe aquisition". At the first glance, these two seem to be unrelated. However, it is known that oxygen induces stress and results in enormous changes in *E. coli *[44,45]. *E. coli *adapts to this environmental change by switching from aerobic respiration (which is its preferred metabolic mode) to anaerobic respiration. More detailed examination of these predictions will be needed to definitively evaluate performance.

Our predictions for ORFs of unknown function (in 2001) are available online at [46]. In this website, we also provide the combined dataset for *E. coli*, and the MATLAB programs for MNL, treeMNL and corMNL along with their respective outputs for the test set.

## Conclusion

In this paper, we investigated the use of hierarchical classification schemes to perform functional annotation of genes. If the hierarchy provides any information regarding the structure of gene function, we would expect this additional information to lead to better prediction of classes. To examine this idea, we compared three Bayesian models: a non-hierarchical MNL model, a hierarchical model based on nested MNL, referred to as treeMNL, and our new corMNL model, which is a form of the multinomial logit model with a prior that introduces correlations between the parameters of nearby classes. We found corMNL provided better predictions in most cases. Moreover, we introduced a new approach for combining different sources of data. In this method, we use separate scale parameters for each data source in order to allow their corresponding coefficients have appropriately different variances. This approach provided better predictions compared to other methods.

While our emphasis in this paper was on the importance of using hierarchical schemes in gene classification, we also showed that even the non-hierarchical Bayesian MNL model outperforms previous methods that used the C5 algorithm. Overall, our results are encouraging for the prospect of accurate gene function annotation, and also illustrate the utility of a Bayesian approach with problem-specific priors. For our experiments, we used the pre-processed datasets provided by [3], who used the Warmr [41] algorithm to generate binary attributes. It is conceivable that the accuracy of predictions can be further improved by using other data processing methods. Similarly, it is possible that a method other than our use of PCA might be better for reducing dimensionally before doing classification.

In the *E. coli *dataset we used here, each ORF was assigned to only one function. In the more recent classification system provided by Riley's group [43], ORFs may belong to more than one class. For such problems, one can modify the likelihood part of the models described here so that if a training case belongs to several classes, its contribution to the likelihood is calculated based on the sum of probabilities of those classes.

The functional hierarchies considered here are simple tree-like structures. There are other hierarchical structures that are more complex than a tree. For example, one of the most commonly used gene annotation schemes, known as Gene Ontology (GO), is implemented as a directed acyclic graph (DAG). In this structure a node can have more than one parent. Our method, as it is, cannot be applied to these problems, but it should be possible to extend the idea of summing coefficients along the path to the class in order to allow for multiple paths.

Our approach can also be generalized to problems where the relationship among classes can be described by more than one hierarchical structure. For these problems, different hyperparameters can be used for each hierarchy and predictions can be made by summing the parameters in branches from all these hierarchies.

## Methods

**Figure 1:**
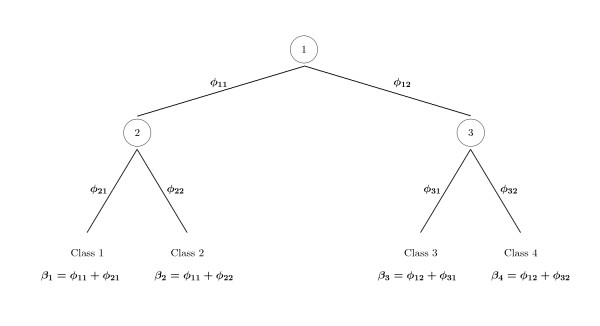
**The corMNL model for a simple hierarchy**. The coeffcient parameter for each class is a sum of parameters at different levels of the hierarchy.

In this section, we first explain our models using a simple hierarchy for illustration. Consider Figure 1, which shows a hierarchical classification problem with four classes. By ignoring the hierarchy, a simple multinomial logit (MNL) can be used for classifying cases to one of the four classes. If the class for a case is denoted by *y*, and the covariates for this case are *x*, then the MNL model is

P(y=j|x,α,β)=exp⁡(αj+xβj)∑j′=14exp⁡(αj′+xβj′)
 MathType@MTEF@5@5@+=feaafiart1ev1aaatCvAUfKttLearuWrP9MDH5MBPbIqV92AaeXatLxBI9gBaebbnrfifHhDYfgasaacH8akY=wiFfYdH8Gipec8Eeeu0xXdbba9frFj0=OqFfea0dXdd9vqai=hGuQ8kuc9pgc9s8qqaq=dirpe0xb9q8qiLsFr0=vr0=vr0dc8meaabaqaciaacaGaaeqabaqabeGadaaakeaacqWGqbaucqGGOaakcqWG5bqEcqGH9aqpcqWGQbGAcqGG8baFcqWG4baEcqGGSaaliiGacqWFXoqycqGGSaalcqWFYoGycqGGPaqkcqGH9aqpdaWcaaqaaiGbcwgaLjabcIha4jabcchaWjabcIcaOiab=f7aHnaaBaaaleaacqWGQbGAaeqaaOGaey4kaSIaemiEaGNae8NSdi2aaSbaaSqaaiabdQgaQbqabaGccqGGPaqkaeaadaaeWaqaaiGbcwgaLjabcIha4jabcchaWjabcIcaOiab=f7aHnaaBaaaleaacuWGQbGAgaqbaaqabaGccqGHRaWkcqWG4baEcqWFYoGydaWgaaWcbaGafmOAaOMbauaaaeqaaOGaeiykaKcaleaacuWGQbGAgaqbaiabg2da9iabigdaXaqaaiabisda0aqdcqGHris5aaaaaaa@601F@

For each class *j *(for *j *= 1, ..., 4), there is an intercept *α*_*j *_and a vector of *p *unknown parameters ***β***_*j*_, where *p *is the number of covariates in *x*. The inner product of these parameters with the covariate vector is shown as *x****β***_*j*_.

Alternatively, we can use the hierarchy to decompose the classification model into nested models (e.g., MNL). For example, in Figure 1, class 1 can be modeled as the product of two independent MNL models:

*P *(*y *= 1|*x*) = *P *(*y *∈ {1, 2}|*x*) × *P *(*y *∈ {1}|*y *∈ {1, 2}, *x*)

We refer to models in which the tree structure is used to define a set of nested MNL models as treeMNL.

For modelling hierarchical classes, we propose a Bayesian MNL with a prior that introduces correlations between the parameters of nearby classes. Our model, called corMNL, includes a vector of parameters, ***φ***, for each branch in the hierarchy (Figure 1). We assign objects to one of the end nodes using an MNL model whose regression coefficients for class *j *are represented by the sum of the parameters for all the branches leading to that class. Sharing of common parameters (from common branches) introduces prior correlations between the parameters of nearby classes in the hierarchy. This way, we can better handle situations in which these classes are hard to distinguish. In Figure 1, parameter vectors denoted as ***φ***_**11 **_and ***φ***_**12 **_are associated with branches in the first level, and ***φ***_**21**_, ***φ***_**22**_, ***φ***_**31 **_and ***φ***_**32 **_with branches in the second level. We assign objects to one of the end nodes using an MNL model with regression coefficients ***β***_**1 **_= ***φ***_**11 **_+ ***φ***_**21**_, ***β***_**2 **_= ***φ***_**11 **_+ ***φ***_**22**_, ***β***_**3 **_= ***φ***_**12 **_+ ***φ***_**31 **_and ***β***_**4 **_= ***φ***_**12 **_+ ***φ***_**32 **_for classes 1, 2, 3 and 4 respectively. Note that the intercept parameters, *α*_*j*_, are not treated hierarchically.

We first used these models (i.e., MNL, treeMNL and corMNL) to predict gene function using each data source (SIM, STR and SEQ) separately. Since the numbers of covariates in these datasets are large, we applied Principal Component Analysis (PCA). Prior to applying PCA, the variables were centred to have mean zero, but they were not rescaled to have variance one. We selected the first *p *components with the highest eigenvalues. The cutt-off, *p*, was set based on the plot of eigenvalues against PCs (i.e., the scree plot). Since there was not a clear cut-off point at which the magnitude of eigenvalues drops sharply, the plots could only help us to narrow down the appropriate values for *p*. We decided to choose a value at the upper end of the range suggested by the scree plot. We selected 100 components from SEQ, 100 components from STR, and 150 components from SIM.

Principal components are derived solely based on the input space and do not necessarily provide the best set of variables for predicting the response variable. In order to find the relevant variables (among the principal components) for the classification task, we use the Automatic Relevance Determination (ARD) method suggested by [47]. ARD employs a hierarchical prior to determine how relevant each covariate is to classification. In the MNL model, for example, one hyperparameter, *σ*_*l*_, is used to control the variance of all coefficients, *β*_*jl *_(*j *= 1, ..., *c*), for covariate *x*_*l*_. If a covariate is irrelevant, its hyperparameter will tend to be small, forcing the coefficients for that covariate to be near zero. We also use a set of hyperparameters, *τ*_*j*_, to control the magnitude of the *β*'s for each class. We use a third hypeparameter, *ξ*, to control the overall magnitude of all *β*'s. This way, *σ*_*l *_controls the relevance of covariate *x*_*l *_compared to other covariates, *τ*_*j *_controls the usefulness of covariates in identifying class *j*, and *ξ *controls the overall usefulness of all covariates in separating all classes. The standard deviation of *β*_*jl*_is therefore equal to *ξτ*_*j*_*σ*_*l*_.

For the MNL model we used the following priors:

*α*_*j*_|*η*~*N*(0, *η*^2^)

*β*_*jl*_|*ξ*, *σ*_*l*_, *τ *~ *N *(0, *ξ*^2 ^τj2σl2
 MathType@MTEF@5@5@+=feaafiart1ev1aaatCvAUfKttLearuWrP9MDH5MBPbIqV92AaeXatLxBI9gBaebbnrfifHhDYfgasaacH8akY=wiFfYdH8Gipec8Eeeu0xXdbba9frFj0=OqFfea0dXdd9vqai=hGuQ8kuc9pgc9s8qqaq=dirpe0xb9q8qiLsFr0=vr0=vr0dc8meaabaqaciaacaGaaeqabaqabeGadaaakeaaiiGacqWFepaDdaqhaaWcbaGaemOAaOgabaGaeGOmaidaaOGae83Wdm3aa0baaSqaaiabdYgaSbqaaiabikdaYaaaaaa@353C@)

*log*(*η*) ~ *N*(0, 1)

*log*(*ξ*) ~ *N*(-3, 2^2^)

*log*(*τ*_*j*_) ~ *N*(-1, 0.5^2^)

*log*(*σ*_*l*_) ~ *N*(0, 0.3^2^)

Since the task of variable selection is mainly performed through PCA, the ARD hyperparameters, *τ*'s, are given priors with fairly small standard deviation. The priors for *τ*'s are set such that both small values (i.e., close to zero) and large values (i.e., close to 1) are possible. The overall scale of these hyperparameters is controlled by *ξ*, which has a broader prior. Note that since these hyperparameters are used only in the combination *ξτ*_*j*_*σ*_*l*_, only the sum of the means for *log*(*ξ*), *log*(*τ*_*j*_), and *log*(*σ*_*l*_) really matters.

Similar priors are used for the parameters of treeMNL and corMNL. For these two models, we again used one hyperparamter, *σ*_*l*_, to control all parameters (*β*'s in treeMNL, *φ*'s in corMNL) related to covariate *x*_*l*_. We also used one scale parameter *τ*_*k *_for all parameters related to branch *k *of the hierarchy. The overall scale of all parameters is controlled by one hyperparameter *ξ*. When we combine different sources of information, we sometimes used separate scale parameters, *ξ*, for each data source. This allows the coefficients from different sourcecs of data to have appropriately different variances in the model. This is additional to what ARD hyperparameters provide.

The setting of priors described in this paper is different from what we used in a previous paper [37], where we used one hyperparameter to control all the coefficients (regardless of their corresponding class) in the MNL model, and we used one hyperparameter to control the parameters of all the branches that share the same node in treeMNL and corMNL. The scheme used in this paper provides an additional flexibility to control *β*'s. In this paper, the hyperparameters are given log-normal distributions instead of the gamma distributions used in [37]. Using gamma priors has the advantage of conjugacy and, therefore, easier MCMC sampling. However, we prefer log-normal distributions since they are more convenient for formalizing our prior beliefs.

## Implementation

These models are implemented using Markov chain Monte Carlo [48]. We use Hamiltonian dynamics [48] for sampling from the posterior distribution of coefficients (with hyperparameters temporarily fixed). The number of leapfrog steps was set to 50. The stepsizes were set dynamically at each iteration, based on the current values of the hyperparameters [47]. In the MNL and corMNL models, new values are proposed for all regression parameters simultaneously. Nested MNL models in treeMNL are updated separately since they are regarded as independent models. The coefficient parameters within each nested model, however, are updated at the same time.

We use single-variable slice sampling [49] to sample from the posterior distribution of hyperparameters. At each iteration, we use the "stepping out" procedure to find the interval around the current point and the "shrinkage" procedure for sampling from the interval. The initial values of the ARD hyperprameters, *σ*'s, were set to the inverse of the standard deviation of their corresponding covariates. The initial values of *τ*'s and *ξ *were set to 1.

Convergence of the Markov chain simulations was assessed from trace plots of hyperparameters. We ran each chain for 5000 iterations, of which the first 1000 were discarded. Simulating the Markov chain for 10 iterations took about 2 minutes for MNL, 1 minute for treeMNL, and 3 minutes for corMNL, using a MATLAB implementation on an UltraSPARC III machine.

## Authors' contributions

RN conceived the study, participated in its design and helped to draft the manuscript. BS participated in the design of the study, performed the statistical analysis, and drafted the manuscript. All authors read and approved the final manuscript.

## Supplementary Material

Additional file 1 **Comparison of direct functional annotaion of several ORFs (whos function was unknown in 2001) with predicted functions using our corMNL model**. For each ORF, we provide its Blattner number, predicted hierarchical class (based on the older hierarchy of *E. coli*), and the corresponding class labels in the first line. The subsequent lines (in italic format) show the recent annotation of each ORF based on direct experiment. Here, SE = "Some Evidence" and NE = "No Evidence". Click here for file

## References

[B1] Altschul SF, Madden TL, Schaffer AA, Zhang J, Zhang Z, Miller W, Lipman DJ (1997). Gapped BLAST and PSI-BLAST: a new generation of protein database search programs. Nucleic Acids Research.

[B2] Pearson WR, Lipman DJ (1988). Improved tools for biological sequence comparison. Proceedings of the National Academy of Sciences (USA).

[B3] King RD, Karwath A, Clare A, Dehaspe L (2001). The utility of different representations of protein sequence for predicting functional class. Bioinformatics.

[B4] DeRisi J, Iyer V, Brown P (1997). Exploring the metabolic and genetic control of gene expression on a genomic scale. Science.

[B5] Eisen M, Spellman P, Brown P, Botstein D (1998). Cluster analysis and display of genome-wide expression patterns. Proceedings of the National Academy of Sciences (USA).

[B6] Brown M, Nobel GW, Lin D, Cristianini N, Walsh SC, Furey T, Ares MJ, Haussler D (2000). Knowledge-based analysis of microarray gene expression data by using support vector machines. Proc Natl Acad Sci USA.

[B7] Marcotte EM, Pellegrini M, Ng HL, Rice DW, Yeates TO, Eisenberg D (1999). Detecting protein function and protein-protein interactions from genome sequences. Science.

[B8] Deng M, Zhang K, Mehta S, Chen T, Sun F (2003). Prediction of protein function using protein-protein interaction data. Journal of Computational Biology.

[B9] Eisen JA (1998). Phylogenomics: Improving functional prediction for uncharacterized genes by evolutationary analysis. Genome Research.

[B10] Rost B (2002). Enzyme function less conserved than anticipated. Journal of Molecular Biology.

[B11] Sjölander K (2004). Phylogenomics inference of protein molecular function: Advances and challenges. Bioinformatics.

[B12] Engelhardt BE, Jordan MI, Muratore KE, Brenner SE (2005). Protein molecular function prediction by Bayesian phylogenomics. PLoS Computational Biology.

[B13] Pavlidis P, Weston J (2001). Gene functional classification from heterogeneous data. Proceedings of the 5th International Conference on Computational Modelcular Biology (RECOMB).

[B14] Deng M, Chen T, Sun F (2004). An integrated probabilistic model for functional prediction of proteins. Journal of Computational Biology.

[B15] Schoikowski B, Uetz P, Fields S (2000). A network of protein-protein interaction in yeast. Nature Biotechnology.

[B16] Rison S, Hodgman TC, Thornton JM (2000). Comparison of functional annotation schemes for genomes. Functional and Integrative Genomics.

[B17] Riley M (1993). Functions of the gene products of Escherichia coli. Microbiology Review.

[B18] IUBMB (1992). Enzyme nomenclature: recommendations of the Nomenclature Committee of the International Union of Biochemistry and Molecular Biology.

[B19] Sattath S, Tversky A (1977). Additive similarity trees. Psychometrika.

[B20] Fox J (1997). Applied Regression Analysis Linear Models and Related Methods Sage.

[B21] Koller D, Sahami M (1997). Hierarchically classifying documents using very few words. Proceedings of the 14th International Conference on Machine Learning (ICML).

[B22] Goodman J (2001). Classes for fast maximum entropy training. Proceedings of the IEEE International Conference on Acoustics Speach and Signal Processing (ICASSP) IEEE press.

[B23] Weigend AS, Wiener ED, Pedersen JO (1999). Exploiting hierarchy in text categorization. Information Retrieval.

[B24] McCallum A, Rosenfeld R, Mitchell T, A N (1998). Improving text classification by shrinkage in a hierarchy of classes. Proceedings of the International Conference on Machine Learning (ICML).

[B25] Dumais ST, Chen H (2000). Hierachical classification of Web content. Proceedings of the 23rd ACM International Conference on Research and Development in Information Retrieval (SIGIR).

[B26] Dekel O, Keshet J, Singer Y (2004). Large margin hierarchical classification. Proceedings of the 21st International Conference on Machine Learning (ICML).

[B27] Cai L, Hoffmann T (2004). Hierarchical document categorization with Support Vector Machines. ACM 13th Conference on Information and Knowledge Management.

[B28] Tsochantaridis I, Hoffmann T, Joachims T, Altum Y (2004). Support Vector Machine learning for independent and structured output spaces. Proceedings of the 21st International Conference on Machine Learning (ICML).

[B29] Cesa-Bianchi N, Gentile C, Zaniboni L (2006). Incremental Algorithms for Hierarchical Classification. Journal of Machine Learning Research.

[B30] Eisner R, Poulin B, Szafron D, Lu P, R G (2005). Improving Protein Function Prediction using the Hierarchical Structure of the Gene Ontology. IEEE Symposium on Computational Intelligence in Bioinformatics and Computational Biology.

[B31] Barutcuoglu Z, Schapire RE, Troyanskaya OG (2006). Hierarchical multi-label prediction of gene function. Bioinformatics.

[B32] Caruana R (1997). Multitask Learning. Machine Learning.

[B33] Zhang J, Ghahramani Z, Yang Y (2005). Learning Multiple Related Tasks using Latent Independent Component Analysis. Proceedings of NIPS 2005 Vancouver Canada (to appear).

[B34] Clare A, King RD (2003). Predicting gene function in Saccharomyces cerevisiae. Proceedings of the European Conference on Computational Biology (ECCB 2003), September 27–30, Paris France.

[B35] Blockeel H, Bruynooghe M, Dzeroski S, Ramon J, Struyf J (2002). Hierarchical multi-classification with predictive clustering trees in functional genomics. Proceedings of the ACM SIGKDD 2002 Workshop on Multi- Relational Data Mining (MRDM 2002).

[B36] Struyf J, Dzeroski S, Blockeel H, Clare A, Bento C, Cardoso A, Dias G (2005). Hierarchical multi-classification with predictive clustering trees in functional genomics. Proceedings Lecture Notes in Computer Science.

[B37] Shahbaba B, Neal RM (2005). Improving classification when a class hierarchy is available using a hierarchy-based prior. Tech Rep 0510 Department of Statistics University of Toronto.

[B38] Blattner FR, Plunkett Gr, Bloch CA, Perna NT, Burland V, Riley M, Collado-Vides J, Glasner JD, Rode CK, Mayhew GF, Gregor J, Davis NW, Kirkpatrick HA, Goeden M, Rose DJ, Mau B, Shao Y (1997). The complete genome sequence of Escherichia coli K-12. Science.

[B39] Riley M, Labedan B, Neidhardt FN, Curtiss RI, Lin ECC, Ingraham JL, Low KB, Magasanik B, Reznikoff W, Riley M, Schaechter M, Umbarger E (1996). E. coli gene products: physiological functions and common ancestries. Escherichia coli and Salmonella: cellular and molecular biology.

[B40] King RD, Wise PH, Clare A (2004). Confirmation of data mining based predictions of protein function. Bioinformatics.

[B41] Dehaspe L, Toivonen H, King RD, Agrawl R, Stolorez P, Piatetsky-Shapiro G, Menlo Park (1998). Finding frequent substructures in chemical compounds. Proceedings of the Fourth International Conference on Knowledge Discovery and Data Minging.

[B42] University of Wales, Aberystwyth, Computational biology group. http://www.aber.ac.uk/~dcswww/Research/bio/ProteinFunction.

[B43] GenProtEC, E coli genome and protome database. http://genprotec.mbl.edu/.

[B44] Spiro S, Guest JR (1991). Adaptive responses to oxygen limitation in Escherichia coli. Trends in Biochemical Sciences.

[B45] Guest JR, Green J, Irvine AS, Spiro S, Lin, ECC, Lynch, AS (1996). The FNR modulon and FNR-regulated gene expression in Regulation of gene expression in Escherichia coli.

[B46] Computer programs, data and results for E coli. http://www.utstat.utoronto.ca/~babak/#ecoliResults.

[B47] Neal RM (1996). Bayesian Learning for Neural Networks.

[B48] Neal RM (1993). Probabilistic Inference Using Markov Chain Monte Carlo Methods Technical Report CRG-TR-93-1, Department of Computer Science, University of Toronto.

[B49] Neal RM (2003). Slice sampling. Annals of Statistics.

