# Supplementary material for: Gene function classification using Bayesian models with hierarchy-based priors
Source: BMC Bioinformatics. 2006 Oct 12;7:448. doi: 10.1186/1471-2105-7-448 (PMC1618412; doi:10.1186/1471-2105-7-448)
Supplement: Additional file 1 — Comparison of direct functional annotaion of several ORFs (whos function was unknown in 2001) with predicted functions using our corMNL model. For each ORF, we provide its Blattner number, predicted hierarchical class (based on the older hierarchy of E. coli), and the corresponding class labels in the first line. The subsequent lines (in italic format) show the recent annotation of each ORF based on direct experiment. Here, SE = "Some Evidence" and NE = "No Evidence". [file 1471-2105-7-448-S1.pdf]

Evidence	bNumber	Class	Class description
SE	b0805	4.1.3	Structural elements > Cell envelop > Outer membrane constituents<br>*Cell structure > Membrane*<br>*Location of gene products > Outer membrane*
SE	b1519	3.2.8	Metabolism of small molecules > Biosynthesis of cofactors, carriers > Menaquinone, ubiquinone<br>*Metabolism > Central intermediary metabolism > Unassigned reversible reactions*
SE	b1533	1.5.2	Cell processes > Transport/binding proteins > ABC superfamily (membrane)<br>*Transport > Electrochemical potential driven transporters > Porters (Uni-, Sym- and Antiporters) > The Major Facilitator Superfamily (MFS)*<br>*Cell structure > Membrane*
SE	b1981	1.5.21	Cell processes > Transport/bindingproteins > MFSfamily<br>*Transport > Electrochemical potential driven transporters > Porters (Uni-, Sym- and Antiporters) > The Major Facilitator Superfamily (MFS)*
SE	b2210	3.4.3	Metabolism of small molecules > Degradation of small molecules > Carbon compounds<br>*Metabolism > Energy metabolism (carbon) > Tricarboxylic acid cycle*
SE	b3839	1.5.2	Cell processes > Transport/binding proteins > ABC superfamily (membrane)<br>*Transport > Cell Substrate transported > Protein*<br>*Cell structure > Membrane*
SE	b1822	2.2.1	Macromolecule metabolism > Macromolecule synthesis, modification > Amino acyl tRNA syn; tRNA modification<br>*Information transfer > RNA related > Modification*
SE	b3223	3.4.3	Metabolism of small molecules > Degradation of small molecules > Carbon compounds<br>*Metabolism > Central intermediary metabolism > Amino sugar conversions*
SE	b3337	3.5.2	Metabolism of small molecules > Energy metabolism, carbon > Anaerobic respiration<br>*Cell processes > Adaptation to stress > Fe aquisition*
SE	b3569	3.4.3	Metabolism of small molecules > Degradation of small molecules > Carbon compounds<br>*Metabolism > Carbon compound utilization > Carbohydrate degradation*
SE	b3955	4.1.3	Structural elements > Cell envelop > Outer membrane constituents<br>*Cell structure > Membrane*
SE	b3222	3.4.3	Metabolism of small molecules > Degradation of small molecules > Carbon compounds<br>*Metabolism > Central intermediary metabolism > Amino sugar conversions*
SE	b0570	6.1.1	Global functions > Global regulatory functions<br>*Regulation > Type of regulation > Transcriptional level*
SE	b0619	6.1.1	Global functions > Global regulatory functions<br>*Regulation > Type of regulation > Transcriptional level*
SE	b2219	6.1.1	Global functions > Global regulatory functions<br>*Regulation > Type of regulation > Transcriptional leve*
SE	b0505	3.3.15	Metabolism of small molecules > Central intermediary metabolism > Pool, multipurpose conversions of intermed. met_m<br>*Metabolism > Central intermediary metabolism > Allantoin assimilation*
SE	b0508	3.4.3	Metabolism of small molecules > Degradation of small molecules > Carbon compounds<br>*Metabolism > Central intermediary metabolism*
SE	b0662	3.5.2	Metabolism of small molecules > Energy metabolism, carbon > Anaerobic respiration<br>*Metabolism > Energy metabolism (carbon) > Aerobic respiration*
SE	b0789	2.2.7	Macromolecule metabolism > Macromolecule synthesis, modification > Phospholipids<br>*Metabolism > Macromolecule (cellular constituent) biosynthesis > Phospholipid*
SE	b2924	4.1.2	Structural elements > Cell envelop > Murein sacculus, peptidoglycan<br>*Cell structure > Membrane*
SE	b2052	3.3.18	Metabolism of small molecules > Central intermediary metabolism > Sugar-nucleotide biosynthesis, conversions<br>*Metabolism > Macromolecule (cellular constituent) biosynthesis > Colanic acid (M antigen)*
SE	b2889	2.2.3	Macromolecule metabolism > Macromolecule synthesis, modification > DNA - replication, repair, restriction/modification<br>*Metabolism > Building block biosynthesis > Cofactor, small molecule carrier biosynthesis > Isoprenoid*

Evidence	bNumber	Class	Class description
NE	b2392	3.5.2	Metabolism of small molecules > Energy metabolism, carbon > Anaerobic respiration<br>*Transport > Substrate transported > Mn+/H*
NE	b0103	1.5.1	Cell processes > Transport/binding proteins > ABC superfamily (atp_bind)<br>*Metabolism > Building block biosynthesis > Cofactor, small molecule carrier biosynthesis > Coenzyme A*
NE	b2530	3.3.15	Metabolism of small molecules > Central intermediary metabolism > Pool, multipurpose conversions of intermed. met_m<br>*Information transfer > Protein related > Posttranslational modification*
NE	b0162	3.5.2	Metabolism of small molecules > Energy metabolism, carbon > Anaerobic respiration<br>*Regulation > Genetic unit regulated > Regulon*
NE	b0613	3.4.3	Metabolism of small molecules > Degradation of small molecules > Carbon compounds<br>*Information transfer --> Protein related*
NE	b2972	3.4.3	Metabolism of small molecules > Degradation of small molecules > Carbon compounds<br>*Information transfer > Protein related > Export, signal peptide cleavage*
NE	b0053	2.1.1	Macromolecule metabolism > Macromolecule degradation > Degradation of DNA<br>*Information transfer > Protein related > Chaperone, folding*
NE	b0441	1.7.1	Cell processes > Cell division<br>*Information transfer > Protein related > Chaperone, folding*
NE	b1199	1.5.23	Cell processes > Transport/binding proteins > Mechanism not stated<br>*Metabolism > Central intermediary metabolism > Unassigned reversible reactions*
NE	b3836	4.2.2	Structural elements > Ribosome constituents > Ribosomal proteins - synthesis, modificationRiboso<br>*Cell structure > Membrane*<br>*Location of gene products > Inner membrane*
NE	b3838	5.1.2	Extrachromosomal > Laterally acquirred elements > Phage-related functions and prophages<br>*Cell structure > Membrane*<br>*Location of gene products > Inner membrane*
